# Baby Steps - An Online Program Promoting the Well-Being of New Mothers and Fathers: A Study Protocol

**DOI:** 10.2196/resprot.5706

**Published:** 2016-07-01

**Authors:** Kyra Hamilton, David Kavanagh, Jennifer Connolly, Leigh Davis, Jane Fisher, Kim Halford, Leanne Hides, Jeannette Milgrom, Heather Rowe, Davina Sanders, Paul A Scuffham, Dian Tjondronegoro, Anne Walsh, Katherine M White, Anja Wittkowski

**Affiliations:** ^1^ Menzies Health Institute Queensland Griffith University Brisbane Australia; ^2^ Centre for Children's Health Research Institute of Health and Biomedical Innovation Queensland University of Technology Brisbane Australia; ^3^ Australian Catholic University Brisbane Australia; ^4^ Jean Hailes Research Unit School of Public Health and Preventive Medicine Monash University Melbourne Australia; ^5^ The University of Queensland Brisbane Australia; ^6^ School of Psychological Sciences University of Melbourne Melbourne Australia; ^7^ Parent-Infant Research Institute Melbourne Australia; ^8^ Institute of Health and Biomedical Innovation Queensland University of Technology Brisbane Australia; ^9^ School of Psychological Sciences University of Manchester Manchester United Kingdom

**Keywords:** Perinatal, Wellbeing, Fathers, Mothers, Online Intervention, Randomized Controlled Trial, Quality of Life, Mental Health

## Abstract

**Background:**

Parental well-being can be seriously impacted during the challenging perinatal period. Most research and support services focus on perinatal psychopathology, leaving a need for programs that recognize and enhance the strengths and well-being of parents. Furthermore, fathers have received minimal attention and support relative to mothers, despite experiencing perinatal distress. New parents have limited time and energy to invest in program attendance, and web-based programs provide an ideal platform for delivering perinatal well-being programs. Such programs are globally accessible, available at any time, and can be accessed anywhere with an Internet connection.

**Objective:**

This paper describes the protocol of a randomized controlled trial investigating the effects on first-time parents’ perinatal well-being, comparing two versions of the online program Baby Steps.

**Methods:**

The clinical trial will randomize 240 primiparous mother-father couples to either (1) Babycare, an online information-only program providing tips on selected childcare issues, or (2) Well-being, an online interactive program including all content from the Babycare program, plus parental well-being-focused content with tools for goal-setting and problem solving. Both programs will be supported by short message service (SMS) texts at two, four, seven, and ten weeks to encourage continued use of the program. Primary outcomes will be measures of perinatal distress and quality of life. Secondary outcomes will be couple relationship satisfaction, parent self-efficacy, and social support. Cost-effectiveness will also be measured for each Baby Steps program.

**Results:**

Participant recruitment commenced March, 2015 and continued until October, 2015. Follow-up data collection has commenced and will be completed May, 2016 with results expected in July, 2016.

**Conclusions:**

Perinatal distress has substantial impacts on parents and their infants, with potential to affect later childhood adjustment, relationships, and development. This study aims to test the impact of a highly accessible online program to support parental coping, and maximize the well-being of both parents. By including fathers in the program, Baby Steps has the potential to engage and support this often neglected group who can make a substantial contribution to familial well-being.

**ClinicalTrial:**

Australian & New Zealand Clinical Trials Registry: ANZCTR12614001256662; https://www.anzctr.org.au/ Trial/Registration/TrialReview.aspx?id=367277 (Archived by WebCite at http://www.webcitation.org/6ibUsjFIL)

## Introduction

The perinatal period is challenging for parents, and the changes and demands encountered during this life transition can seriously impair parents’ well-being. The impact of these challenges is well-recognized in mothers with postpartum depression rates of approximately 20% in Australian women [1]. Most new mothers (50-80%) experience some distress [2]. A meta-analysis of fathers’ postpartum mental health found comparable rates of depression (10%) [3], with Australian figures reporting the rate at approximately 5% [4,5]. Initiatives by *beyondblue* and the Federal Government have increased perinatal depression screening and intervention rates in Australia. However, screening and preventive programs for perinatal depression have not been universally available [6], and have been almost non-existent for fathers, despite the potential suitability of the Edinburgh Postnatal Depression Scale (EPDS) for fathers [7]. Lack of availability of these father-specific programs is a reflection of both supply (eg, a focus on mothers, service capacity, and staff adherence) and demand issues (eg, reluctance to disclose symptoms and receive treatment) [8]. An accessible perinatal well-being intervention designed for both mothers and fathers is the target of this trial.

Postpartum distress has significant and wide-ranging consequences. In mothers, postpartum distress affects attachment [9], child development [10], and academic achievement [11]. Some effects can be long-lasting, including increased risks of later childhood behavioral problems [12] and subsequent episodes of parental depression [13]. More recently the negative effects of paternal postpartum distress have been reported, including its association with emotional difficulties in children [14], the impact on marital relationships and family well-being [15], and the detrimental effect on the mother’s relationship with her child [16].

In addition to significant costs to family well-being, perinatal distress has high economic costs. The annual health costs associated with perinatal depression and anxiety could be up to AUD $70 million in new mothers and AUD $16 million in new fathers, if their baby was delivered in 2012 [17]. The cost of psychological distress in otherwise healthy adults is substantial. For example, lost productivity cost Australian employers AUD $5.9 billion in 2009; productivity losses for each treatment seeker were AUD $19,810 for men and AUD $9183 for women [18]. The cost from one year’s lost productivity for untreated maternal depression is AUD $142 million [17].

Many mothers prefer psychosocial treatments during the postpartum period, especially while breastfeeding [19]. Rapid acting, easily communicated treatments for depression in other contexts involve behavioral activation [20], physical activity [21], problem solving [22], parenting skills and self-efficacy [23-25], social support networks [24,26], and meditation mindfulness [27]. Treatment with a mental health professional is not easily accessible for non-urban populations, creating a barrier for treatment [28].

There is growing evidence, however, that interventions may not always require substantial clinician involvement to reduce distress. A single face-to-face psychoeducational session with a maternal and child health nurse was found to reduce depression and anxiety in women without psychiatric histories [23]. Another study found no difference in clinical outcomes between a psychoeducational booklet and group cognitive behavior therapy (CBT) in primary care [29]. In the second study, however, 60% of eligible women turned down group CBT, highlighting that traditional interventions may not match the needs of new parents. Adherence to treatment appeared especially low when symptoms were mild, women had competing commitments, or motivation was limited. Women also cited time, childcare, and transportation as further barriers to engagement.

Online interventions may overcome these uptake barriers, as the Internet is a highly accessible and cost-effective vehicle for the delivery of flexible and sustainable support. In 2012-2013, 83% of Australian households had Internet access [30], and 89% had a mobile phone with Internet access [31], indicating that electronic interventions are available to most Australians. Internet-based interventions have well-established efficacy for alleviating depression and anxiety, and for promoting well-being [32]. Clinical gains approximate those from face-to-face treatment, especially when dealing with low-severity problems [33]. In addition, stigma is avoided and location inequities are minimized.

The Internet has already proven feasible for screening postpartum depression in mothers [34]. Furthermore, randomized controlled trials (RCTs) for two online postpartum depression treatment programs found decreases in postpartum depression at 12 weeks [35] and 17 weeks [36] respectively, as compared to conventional treatment. Online treatment for postpartum depression shows promise, and may address the substantial economic, community, and family impact of perinatal distress. Development of programs that promote the maintenance and enhancement of perinatal mental well-being should be a priority.

The perinatal period is a time of transition [37], offering a window of opportunity when timely introduction of tools and information could positively influence later outcomes [38]. For example, a study investigating the well-being of 5000 mothers found that positive maternal outcomes after birth are influenced by mental and physical well-being during the perinatal period [38]. Perinatal well-being is a complicated construct determined by an individual’s perception and evaluation of their life during the perinatal period [37]. If expectant and new parents were offered tools to support and enhance well-being throughout this process, both maternal [39] and paternal [40] outcomes could be improved *.* However, few universal strategies for preventing perinatal distress and enhancing well-being have been evaluated in rigorous, large-scale studies [37].

Online programs targeting prevention of postpartum depression are in their infancy. A fully-automated program developed for pregnant women presented psychological strategies across 44 short sessions [41]. Information in this program was presented on a schedule, and this lack of flexibility may be a deterrent for some mothers. An RCT testing the effectiveness of this program is currently underway [42]. A second online prevention program has been evaluated in a pilot trial, but the study found no differences in effectiveness between the online program and the information-only control condition [43]. A limitation of this study was the minimal program use by participants, suggesting that email or text message reminders could be used to increase retention and use of the program. Furthermore, the intervention was linear in design, meaning that if earlier program material was not of significant interest to participants, program use would discontinue.

Although some programs addressing the prevention of postpartum depression in women have succeeded in engaging fathers [23,44,45], fathers’ mental health and well-being has not been specifically targeted. Fathers are offered little support in the way of treatment or prevention of perinatal distress. Syntheses of studies investigating men’s experiences ante- and postnatally found that fathers felt overlooked in antenatal classes [46], and excluded and unsupported by maternity services in general [47]. Despite this limitation, fathers reported a desire to be actively involved in this life experience, and to be supportive of their partners [48], indicating that an opportunity to involve invested fathers is being missed. Based on qualitative interviews with new fathers, Kowlessar et al recommend that men are actively engaged, informed, and offered support [48], and that the presentation of psychoeducation material and well-being tips may help fathers feel more involved and valued during this life stage [40]. More research is required, and high quality RCTs of interventions that promote psychological well-being in both mothers and fathers are needed [49].

To date, no study has trialed a web-based intervention targeting perinatal well-being in both mothers and fathers, nor has a related cost-effectiveness analysis been conducted. This proposed study addresses these gaps, and constitutes one of few trials that focus on well-being and universal prevention, rather than the treatment of established depression. Due to this novelty, our results are likely to have wide application. Electronic programs have the potential to alleviate maternal and paternal distress, and help recent parents enjoy the enriching and exciting experience of early parenthood by increasing access to effective support at minimal cost. By addressing parents’ well-being and supporting childcare, participants may also assist in promoting positive parenting, offering potentially long-range benefits for this and future generations of Australian children.

### Trial Aims

The aim of this trial is to conduct the first RCT of a web-based intervention, *Baby Steps*, that targets perinatal well-being in both mothers and fathers (trial registration number ANZCTR12614001256662). Specifically, the first aim is to compare the effectiveness on indices of well-being using two versions of the *Baby Steps* program for first-time parents: *Babycare* (an online information-only program providing information and tips on selected childcare issues) and *Well-being* (an interactive online program including all *Babycare* content, plus parental well-being-focused content with goal-setting and problem solving tools). The second aim is to assess the cost-effectiveness of the *Well-being* program compared to the *Babycare* program.

### Hypotheses

It is predicted that participants receiving the *Well-being* program will show lower levels of distress and a higher level of quality of life at 13 and 26 weeks post-baseline, compared to participants receiving the *Babycare* program. In addition, couple relationship satisfaction, parental self-efficacy, social support, and cost-effectiveness are predicted to be greater at 13 and 26 weeks post-baseline for participants receiving the *Well-being* program, compared to participants receiving the *Babycare* program.

## Methods

### Study Design

**Figure 1:**
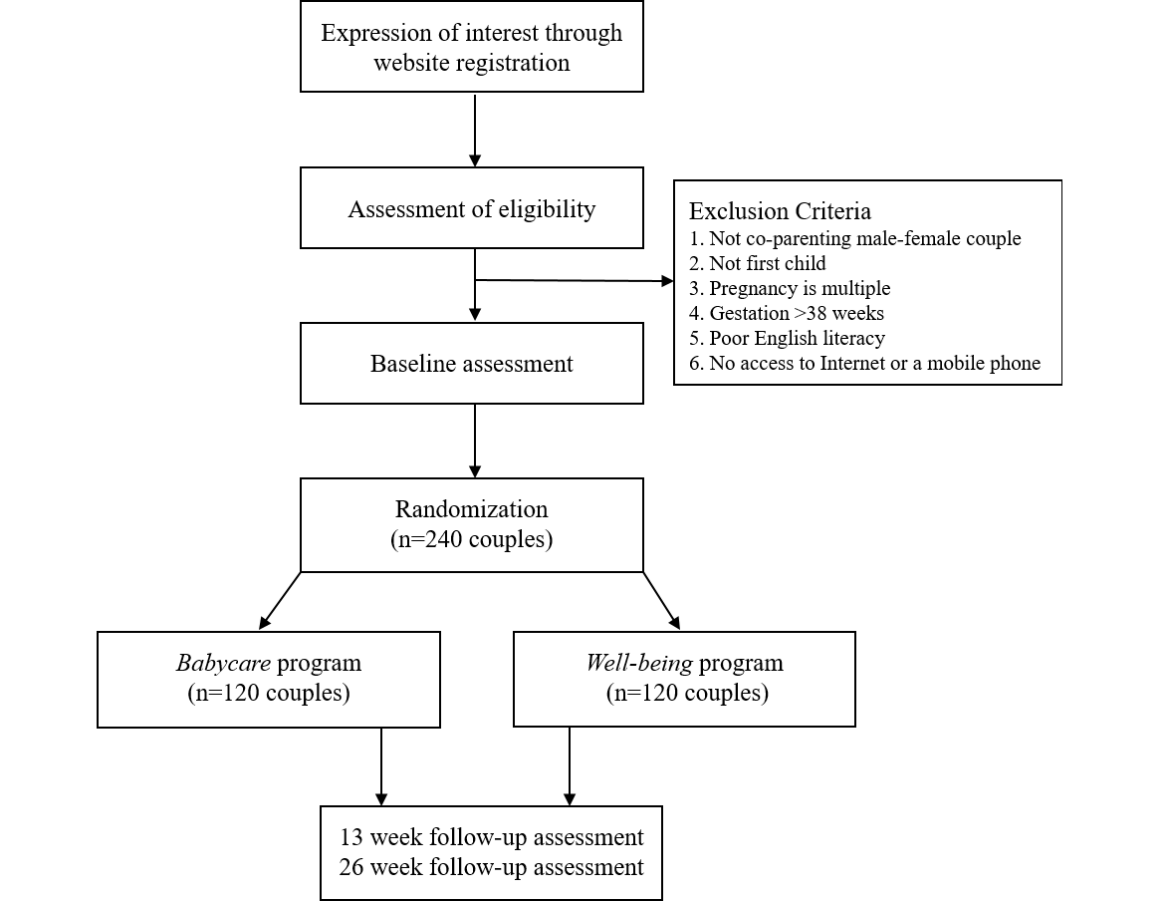
Flowchart of the study design.

This study is an RCT designed to evaluate the effectiveness of an online intervention targeting perinatal well-being in first-time mothers and fathers. After baseline data are collected, participating couples will be randomly allocated to receive either the *Babycare* program or the *Well-being* program. Figure 1 provides a summary of participant flow through the trial.

### Study Sample and Procedure

#### Recruitment Procedures

A total sample of 240 primiparous mother-father couples will be recruited. Participants will primarily be recruited through private and public antenatal parent education classes at the Mater Mothers’ Hospital, Brisbane, Australia. A member of the research team will visit the class immediately before the session commences to inform participants about the study and program. Couples will have the opportunity to ask questions and those interested in participating will be assisted with registration for the study. Participants will also be recruited through an exposition targeting expectant parents, and nationally through advertisements on social media and parenting forums, which will direct couples to the program website. Interested couples will be given the opportunity to ask further questions of the research team via email or telephone. During registration, all participants will be asked to select from a dropdown box regarding how they were first informed about the research trial.

#### Inclusion Criteria

Participants will be included in the trial if they are a co-parenting male-female couple aged 18 years or older, this is the first child for both parents, gestation is between 26 and 38 weeks at registration (couples registering before 26 weeks of gestation will be reminded by email to retake the screening survey when they reach the 26-week time point), and the pregnancy involves a single child. Participants must also be literate in English, have Internet and mobile phone access, and complete the baseline assessment. Both parents must be eligible for the couple to participate. Exclusion criteria are presented in Figure 1.

#### Screening and Baseline

Couples will register for the study by creating a website account for each user with email addresses and passwords. Registrants will then be individually emailed with a link to the screening survey, in which the trial information statement is provided. Participants will confirm that they have read and understood the statement, and that they consent to participate in the research before continuing. Participants will then complete the screening survey to confirm that they meet the study inclusion criteria. Eligible participants will be directed to the online baseline assessment survey, and both parents must complete the survey before the couple is randomly assigned to an intervention condition. Registrants who are not eligible for the study will still be able to access the *Babycare* program.

Reminder emails will be sent to registered participants on an automated schedule if they or their partner have not completed the screening survey three days post-registration, or the baseline survey three days post-screening. If the survey is still not complete 10 days post-registration, participants will be contacted by a research officer to assist with completion of the survey (if the couple gives ongoing consent to participate). If both individuals have not completed the baseline assessment four weeks after eligibility is confirmed, couples will be withdrawn from the study and offered access to the *Babycare* program. After completion of the baseline assessment and randomization, couples will be telephoned by a researcher (blind to treatment allocation) to welcome them to the study, collect postal address details, and provide the research team’s contact information.

#### Follow-Up Assessments

Follow-up assessments will be at 13 and 26 weeks post-baseline. For the purposes of these assessments, couples will be treated as individual participants and emailed an individualized online survey link. To maximize retention, participants will be reminded to complete the survey via scheduled emails. If the participant has not responded within two weeks of the due date, a researcher (blind to treatment allocation) will phone the participant, establish ongoing consent from the participant, and attempt to obtain the assessment over the phone. Participants will be offered an AUD $20 gift voucher (AUD $40 per couple) for completing each follow-up assessment.

#### Risk Management

Given the high frequency of perinatal distress, it is expected that some participants will experience distress during the study. Although the program focuses on well-being and does not specifically address depression, participants will be monitored for signs of elevated distress through their responses on the EPDS, and will be followed-up with according to the protocol outlined in Table 1. Researchers who make these calls will be trained in risk assessment procedures, will receive clinical supervision weekly to fortnightly, and will be blind to treatment allocation.

**Table 1:** Risk management protocol after administration of the Edinburgh Postnatal Depression Scale.

Risk level	Risk cut-offs	Action
Low	Women: 0-9 total score Men: 0-5 total score AND 0 on Item 10	No action required.
Medium	Women: 10-12 total score Men: 6-8 total score OR 1 on Item 10	1. Email participant to organize a time to speak on the phone. Call within one week. 2. Re-administer the EPDS and check score against cut-offs. If now low risk, briefly discuss change between previous EPDS and current EPDS, and discuss what to do if situation worsens. Email referral information. If now high risk, follow protocol for high risk. If still medium risk, follow protocol below. 3. Discuss support options (ie, partner, family, friends). 4. Give support and referral information over the phone and by email: - PANDA (Post and Antenatal Depression Association) 1300 726 306 http://www.panda.org.au/ - Lifeline 13 11 14 https://www.lifeline.org.au/Get-Help/ - Mensline 1300 78 99 78 http://www.mensline.org.au/ - Family doctor - Psychologist www.psychology.org.au/FindaPsychologist/Default.aspx?ID=5911
High	Women: >13 total score Men: >9 total score OR 2 or 3 on Item 10	1. Email participant to organize a time to speak on the phone. Call same day. 2. Discuss support options (ie, partner, family, friends). 3. Give support and referral information over the phone and by email: - PANDA (Post and Antenatal Depression Association) 1300 726 306 http://www.panda.org.au/ - Lifeline 13 11 14 https://www.lifeline.org.au/Get-Help/ - Mensline 1300 78 99 78 http://www.mensline.org.au/ - Family doctor - Psychologist www.psychology.org.au/FindaPsychologist/Default.aspx?ID=5911 4. Assist participant to make a plan to access support, including immediate support if needed. 5. Arrange to speak again in one week, at which time the EPDS will be re-administered, and check score against cut-offs. If still high risk, remain engaged with participant until they are engaged with appropriate professional.

#### Randomization

Allocations will be generated by the Goji program, a web-based research trials management system developed at Queensland University of Technology, Australia, by the same web developers who programmed the *Baby Steps* programs. When the researcher notifies Goji that a participating couple has completed their baseline assessment, Goji will allocate the participants to a research group, randomizing couples as a unit. Randomizations will be in permuted blocks, and stratified by scores on the EPDS: neither parent distressed (father <5; mother <7) versus either parent distressed (father >5; mother >7). These cut-off scores represent optimum screening levels for depressive or anxiety disorders in fathers and mothers [7]. After randomization, a couple’s access to the allocated program will be granted by a researcher not blind to treatment allocation, and participants will be notified by email that they are able to log into the online program through their registered account.

#### Dropout

Couples will be randomized as a unit but may elect to withdraw individually or as a couple. An individual will be able to continue participating in the trial if their partner withdraws, and this will not have any effect on their participation in the trial.

#### Sample Size

A total of 240 couples (120 couples per group) will be recruited. A sample size of 240 will give a power of .80 for detecting a significant *Time* x *Condition interaction* (*P*<.05) over three occasions of measurement, with a small effect size of f=0.082 [50,51].

### Online Programs

#### Program Content, Tone, and Design

The content of the *Baby Steps* programs, *Babycare* and *Well-being,* was developed by a team comprised of clinical psychologists, midwives, and a neonatologist. *Baby Steps* is a modular, self-paced program designed to provide infant care and well-being information, and tools relevant for mothers and fathers during late pregnancy until their infant is approximately six months of age. Each module contains 4-8 categories that help to guide program users through the information contained within the module, or information presented through external websites and programs. Suggestions are offered for employing the information and tips individually, as parent and infant, as a couple, or as a family. A *Get Help* tab includes links to other parenting websites and programs, and links to face-to-face, telephone, or online health service providers.

Program information is written in an encouraging and non-judgmental tone, designed to create an empathic environment in which new parents can access information and tools. Inclusive language is used to aid engagement of both mothers and fathers in the program and gender role assumptions have deliberately been avoided when presenting the content. For example, information about returning to work after a brief leave is provided for both mothers and fathers, as are tips and suggestions for helping to feed an infant. Photos and images of both men and women interacting with their infants are used throughout the program.

#### Babycare Program – Control Condition

The *Babycare* program is comprised of four modules: (1) *Getting Prepared* – practical tools to help expectant parents prepare for the arrival of an infant, (2) *Feeding* – information about breastfeeding, formula feeding, and combined feeding, (3) *Improving Baby’s Sleeping Habits* – information for understanding the patterns of infants’ sleep and sleep cues, plus practical tools for creating a sleep routine, and (4) *Soothing* – information and practical techniques to calm a crying infant.

##### Short Message Service Reminders

Automated short message service (SMS) texts will be sent to *Babycare* participants two, four, seven, and ten weeks after program allocation to remind them to log into the program.

#### Well-Being Program – Experimental Condition

*Babycare* is an information-only program, while *Well-being* is an interactive program including the four *Babycare* program modules along with five additional modules promoting parental well-being, and utilizes interactive tools for the enhancement of well-being. The five additional modules are: (1) *Self-Care* – information and tips to assist new parents to look after their physical and mental health, (2) *Relationships* – tools for working through changes in the romantic relationship after an infant is born, (3) *Interacting with Baby* – information to assist new parents to understand, bond with, and play with their infant, (4) *Changing Roles* – tips to assist new parents to adjust to the new roles that parenthood brings, and (5) *Especially for Fathers* – well-being and infant care information targeted specifically to fathers.

After qualitative interviews with mothers and fathers who trialed the *Baby Steps* programs, the *Especially for Fathers* module was developed to provide additional father-targeted information. However, this module is also available to mothers who may wish to understand the role of fathers, and provides useful tips for his well-being.

##### My Plans

Within each module, *Well-being* participants can choose tips to send to the *My Plans* tool, which functions as a goal-setting, problem-solving, and behavioral activation tool. Participants are encouraged to develop plans to incorporate the chosen tips into their lives, including setting a specific date and time when the plan will be attempted, or a regular frequency at which they will complete the plan. Example suggestions for plans that may be attractive to mothers, fathers, or both parents are distributed throughout the modules. A participant using the *Self-Care* module could select the *Talk to someone about what’s stressing you* tip, and choose to develop a plan to *Book an appointment with my General Practitioner* and to complete the plan on *November 10*^th^*.* Participants are able to review and update their plans throughout their use of the program, and can mark plans as completed.

##### Additional Tools

The *Well-being* program also includes a *Scrapbook* tool, in which participants can upload and view photos that remind them of pleasure and success. The participant’s personalized *Dashboard* displays *Scrapbook* photos, upcoming plan due dates, rotating module tips, and light-hearted, infant-specific trivia questions.

##### Short Message Service Reminders

Participants allocated to the *Well-being* program will also receive automated SMS texts two, four, seven, and ten weeks after allocation, reminding them to log into the program, select useful tips, develop plans to enhance well-being, and regularly review the progress of their plans.

### Intervention Integrity

Reporting of this study is guided by the Consolidated Standards of Reporting Trials statement [52] and international best practice. The randomization schedule is concealed within a secure database accessible only to the Goji development manager. Researchers collecting post-baseline assessments will be blinded to condition. Calls made to welcome participants to the study, or to conduct a risk assessment, will be made by a researcher blind to treatment allocation. Data analyses will be conducted based on the principle of intention-to-treat [53].

### Study Measures

Measures will be restricted to minimize the burden on participants. Demographics collected will include details of income and occupational engagement. Economic information including healthcare data, work time/productivity data, and intervention establishment/delivery data will also be collected. Past and recent psychiatric history will also be assessed. Information concerning the outcome of the pregnancy will be collected 13 weeks post-baseline. The EPDS [54] will be used to assess perinatal distress and the four-item Social Support Survey [55] will measure satisfaction with social support. The Assessment of Quality of Life-8 Dimensions (AQol-8D) [56] will be used to assess quality of life, and relationship satisfaction will be measured by the Couples Satisfaction Index [57].

Three measures were designed specifically for this study. These measures were created to be brief, as to minimize time burden on participants, and designed to directly measure certain program components. Self-efficacy over the initial 13 weeks of child-rearing, and providing support to a partner (ie, *feeding your baby*, *putting your baby to sleep*, *settling your baby*, *providing support to your partner*), will be assessed on an 11-point scale. The scale increases in 10-point increments, and ranges from 0 (not at all confident) to 100 (extremely confident). Current satisfaction with participants’ parenting role and skills, and with relationships and social support (ie, *relationship with their partner*, *support received from and given to their partner*, *practical and emotional support received from others*), will be rated on a similar 11-point scale from 0 (not at all satisfied) to 100 (extremely satisfied). The same rating scale will be used for program satisfaction at 13 weeks post-baseline (ie, *overall*, *relevance*, *usefulness*, *how easy it was to find what they wanted*). Timing of the assessments is presented in Table 2.

**Table 2:** Study measures and timing.

Measure	Domain	Baseline	13 week follow-up	26 week follow-up
Demographics	Demographics			
Work history and performance	Recent employment history and performance			
Birth items	Baby and birth details			
AQol-8D	Quality of life/well-being			
Edinburgh Postnatal Depression Scale	Perinatal distress			
Self-efficacy items	Parental self-efficacy			
Satisfaction items	Satisfaction			
Social Support Survey	Social support			
Couples Satisfaction Index	Relationship satisfaction			
Psychiatric history	Psychiatric history			
Recent medical history	Medical history, healthcare data			
Program satisfaction items	Program satisfaction			
Program use	Program engagement			

Both *Baby Steps* programs automatically collect program use data that are summarized into engagement variables (number of logins, module pages viewed, number of plans made, number of photos uploaded to the Scrapbook, and total time spent on the module pages).

### Data Analyses

Version 22 of the IBM SPSS Statistics program will be used to perform all data analyses.

#### Descriptive Analyses

Baseline data from the two program groups will be compared to check for comparability of the groups. Chi-square tests will be used for categorical variables, ANOVA tests for normally distributed continuous variables, and Kruskal-Wallis tests for non-parametric data. Any differences detected will be controlled for during the subsequent analyses.

#### Primary and Secondary Analyses

All data analyses will be based on the intention-to-treat principle [53]. Continuous outcomes (eg, measures of perinatal distress, quality of life, couple relationship satisfaction) will be analyzed using general linear mixed models, and multilevel modelling will be used for nested data, to deal with any missing data.

#### Cost-Effectiveness Analysis

The evaluation will examine the perspectives of costs to health services and to society. Unit costs will be applied to resource-use data collected on health care (primary care and hospital data), work time/productivity, and intervention establishment/delivery. The AQol-8D [56], a multi-attribute utility instrument, will be used and scored using the Australian algorithm [58]. Resultant utility weights will be multiplied by duration to estimate quality-adjusted life years. Incremental cost-utility ratios will be estimated as the primary outcome factor of the economic evaluation. An alongside-trial analysis will be undertaken. Sensitivity analyses will be used to identify key factors driving the results and bootstrapped standard errors will be used to estimate the 95% confidence interval for the incremental cost-effectiveness ratio.

### Ethical Approval

This trial received ethical approval from the Human Research Ethics Committees of Queensland University of Technology (No. 1400000687) and the Mater Health Services (No. HREC/14/MHS/166). The trial will be implemented in compliance with this protocol and the Australian National Statement on Ethical Conduct in Human Research [59].

## Results

Participant recruitment commenced March, 2015 and continued until October, 2015. Follow-up data collection has commenced and will be completed May, 2016 with results expected in July, 2016.

## Discussion

Depression and distress have substantial impacts on new parents and their babies, with potential to affect later adjustment, relationships, and child development [11-16]. The economic impacts of such depression are also large [17,18]. The perinatal period is an opportunity to engage expectant parents in well-being-based programs, which is particularly important given that better well-being during this phase is associated with positive postpartum outcomes [38]. Although mothers are often the focus of support and interventions during the perinatal phase, fathers also experience perinatal distress, but have received little research attention or support from services, despite a desire to be involved during the perinatal period [45]. Internet interventions offer a flexible, accessible, cost-effective solution for enhancing perinatal well-being in mothers and fathers.

The current research addresses several issues. This project has developed an innovative program with a unique combination of modules, based on several related programs designed by the authors. *Baby Steps* screens for distress and depression, provides childcare resources and interactive tools, and addresses emotional and physical well-being of both fathers and mothers. Key foci are self-selected childcare issues and maintaining pleasurable physical and social activity. Personal tailoring, after-hours availability, rural accessibility, and low cost per user allow the platform to be easily disseminated to large numbers of people across Australia. This study conducts the first perinatal trial to differentiate component effects from a combined intervention of this type, examining distress, functioning, and childcare confidence, and is the first to compare cost-effectiveness of such interventions. As such, this project helps to facilitate the translation of research into policy and practice at state and national levels. *Baby Steps* is one of few trials that focuses on well-being and prevention rather than established depression (thereby having wide applicability), and is also one of the few trials to focus on the well-being of new fathers.

This project has the potential to influence standard services to support parents, thereby increasing their effectiveness, with minimal increases in cost. *Baby Steps* offers a strategy to engage and support fathers, improving their own outcomes as well as their support of mothers, and potentially preventing losses in work time and productivity associated with depression. In concert with other strategies to address the causes of depression and anxiety, this project assists in promoting the long-term emotional and physical health and well-being of Australian children and their families.

## Abbreviations

AQol-8D: Assessment of Quality of Life-8 Dimensions
CBT: cognitive behavior therapy
EPDS: Edinburgh Postnatal Depression Scale
RCT: randomized controlled trial
SMS: short message service
